# Development of Loop-Mediated Isothermal Amplification (LAMP) Assays for the Rapid Authentication of Three Swimming Crab Species

**DOI:** 10.3390/foods11152247

**Published:** 2022-07-28

**Authors:** Soottawat Benjakul, Jirakrit Saetang

**Affiliations:** International Center of Excellence in Seafood Science and Innovation, Faculty of Agro-Industry, Prince of Songkla University, Hat Yai 90110, Songkhla, Thailand; soottawat.b@psu.ac.th

**Keywords:** LAMP, PCR, blue swimming crab, crab meat mislabeling, food fraud

## Abstract

Blue swimming crab meat is easily adulterated by other crab meats with a lower price. A potential authentication method is required to prevent mislabeling. LAMP assays were established to identify the meat of blue swimming crab, crucifix crab, and three spotted swimming crab. The primers were designed using PrimerExplorer V5. The specificity of the LAMP assay was tested compared to the PCR method. The sensitivity was conducted at the DNA concentrations of 0.4–50 ng/reaction. The results demonstrated that both LAMP and PCR could discriminate all species of crabs. LAMP showed a superior sensitivity to PCR in the three spotted swimming crab, while a similar result between LAMP and PCR was obtained in blue swimming crab. No changes in the detection efficacy were attained when boiled and steamed crab meats were applied. Therefore, the LAMP assay developed could potentially be applicable to detect the adulteration or mislabeling of raw or cooked crab meat in markets.

## 1. Introduction

Most of the economically important crabs belong to a group of *Portunidae* species, some of which are important for aquaculture and are in high demand [1]. This kind of crab possesses a unique organ that gives them the swimming behavior called 5th pereiopods or P5-swimming [2]. Nowadays, most crabs found in restaurants and seafood markets are from this family because of their scrumptious meat, high nutritional value, good flavor and taste as well as accessibility [3]. In addition, swimming crabs have increasingly gained attention in the world market, especially the crab products from Asian countries [4]. With this increasing demand, certain species of swimming crabs such as the blue swimming crab are recognized worldwide and have become valuable seafood products [5]. This globalization has led to public concern regarding the identity and safety caused by the product mislabeling issue related to adulteration [6].

Blue swimming crab (*Portunus pelagicus*) is a member of the *Portunidae* family, which displays the most dominant species in the crab market. The fisheries and aquaculture of blue swimming crab are mainly found in the West Pacific Ocean, and the globally growing frozen and canned crab meat industry has led to great profits for the fisherman and communities. In 2016, about 260,000 tons of blue swimming crab were captured from a natural source, while aquaculture production yielded around 29 tons, reflecting the popularity of blue swimming crab in the global seafood market [7]. Normally, blue swimming crab products are commercially available in a variety of forms including whole-body products such as live crabs and chilled crabs, or processed forms (i.e., steamed, mixed, frozen, and canned crab meat) [8]. In general, the former form still maintains the visual characteristics or identity of the species. On the other hand, the latter, especially crab meat, cannot be differentiated due to the loss of morphological features. This may cause the chance of the mislabeling of species with the intentional substitution or fraud with lower-valued swimming crab species such as the three spotted swimming crab (*Portunus sanguinolentus*) or crucifix crab (*Charybdis feriata*). Therefore, food authentication methods for species identification are important for tackling the adulteration of other crab meat into blue swimming crab meat.

Pioneering food authentication methods are based on the identification of the unique compounds that discriminate one species from others by using tools that measure the biomolecules such as DNA, proteins, lipids, sugars, and/or metabolites, or inorganic matters (i.e., elements and isotope ratios) as well as the identification of secondary markers of authenticity [9]. With the growth in molecular genetics and DNA technologies, the analytical methods have been modernized and the use of DNA barcode sequences has been adopted without the need of morphological observation [10]. DNA sequences for the authentication of species in seafood demonstrate many benefits since the meat samples, either in their raw or cooked forms, without their identity, can still be used [10]. Moreover, the variability of the DNA data is species- or subspecies specific, allowing for the potential identification of adulterated food [11], especially with decapod crustaceans, in which the average genetic distance between species and intraspecific variation is 17.16% and 0.46%, respectively [12]. This principle has been considered as an effective tool for seafood authentication, in which the development of the Reference Standard Sequence Library (RSSL) for seafood identification is emphasized. This brings about the guidance of using DNA sequences in seafood authentication with certain specimens [13].

Nowadays, a wide range of molecular genetic techniques has been employed for seafood authentication such as Sanger’s sequencing of the DNA barcode [14], multiplex PCR [15], and PCR-based restriction fragment length polymorphism (PCR-RFLP) analysis [16]. Although the PCR-RFLP method is quite popular because it is a cheaper alternative to other PCR-based protocols [16,17], the requirement of different restriction enzymes for different species and the need for high-resolution agarose gel to resolve the close size of restriction products is still an obstacle or limitation [18]. Moreover, a thermocycler is also essential for all PCR-based techniques, which may not be practical for all laboratories. Alternatively, loop-mediated isothermal amplification (LAMP) is a novel adulteration detection method that requires basic simple reagents and instruments including *Bst* DNA polymerase, two primer pairs, and a water bath or heat block for reaction [19]. This technology provides the high specificity resulting from the use of two pairs of primers (F3, B3, FIP, and BIP) that recognize six regions of the DNA target (F1c, F2c, F3c, B2c, B1c, and B3c). The *Bst* polymerase contains both strand displacement and DNA polymerase activities under the isothermal temperature and the reaction can proceed using a portable temperature controlled machine such as a water bath or heat block [20]. These advantages make the LAMP method as attractive and suitable for food authentication, especially in seafood products.

Since blue swimming crab meat has been normally mixed with cheaper species such as three spotted swimming crab and crucifix crab, etc., a potent authentication method is required. In the present study, three sets of primers were designed based on the highly conserved mitochondrial gene, cytochrome C oxidase (COI), with specificity to each species. The sensitivity of the developed method toward both raw and cooked crab meat was also examined. Finally, the conventional PCR technique using the F3 and B3 primers was also conducted for a comparison.

## 2. Materials and Methods

### 2.1. Raw and Cooked Samples

Three species of swimming crabs including blue swimming crab (*Portunus pelagicus*), three spotted swimming crab (*Portunus sanguinolentus*), and crucifix crab (*Charybdis feriata*) were purchased from local fishermen (Songkla, Thailand). For each species, the lump meat was removed from the shell manually after thawing overnight at 4 °C. The collected meat was placed in a zip lock sterile plastic bag and stored at −80 °C until use. For the boiled crab meat, the meat packaged in sealed plastic bags was boiled at 85 °C for 5, 10, and 15 min in a controlled water bath (Esstell, South Korea). Steamed samples were prepared by heating whole-body crabs at 105 °C in a steaming pot for 20, 30, and 40 min. The meat of the steamed crabs was collected manually and placed in a sterile Ziplock bag. All meat samples were stored at 4 °C for less than two days before analysis.

### 2.2. DNA Extraction and Quality Evaluation

DNA was extracted from at least 30 mg of raw and/or cooked samples using the PureLink™ Genomic DNA Mini Kit (Invitrogen, Waltham, MA, USA) according to the manufacturer’s instructions. The DNA quality and quantity were measured using NanoDrop^®^2000 (Thermo-Scientific, Waltham, MA, USA) and stored at −20 °C until use.

### 2.3. LAMP Primer Design

All LAMP primers (F3, B3, LoopF, LoopB, and/or FIP and BIP) were designed using PrimerExplorer V5 online software (http://primerexplorer.jp/lampv5e/index.html (accessed on 17 April 2022); Eiken Chemical Co. Ltd., Tokyo, Japan) based on the sequence of the cytochrome c oxidase subunit I (COI or COX1) gene of each kind of swimming crab. All sequence data were retrieved from the GenBank database (http://www.ncbi.nlm (accessed on 17 April 2022). The accession numbers of the COI gene for blue swimming crab (*Portunus pelagicus*), three spotted swimming crab (*Portunus sanguinolentus*), and crucifix crab (*Charybdis feriata*) are 22909203, 26121718, and 20004274, respectively. The details of each primer set are demonstrated in Table 1.

### 2.4. LAMP Reaction

All LAMP reactions (25-µL volume) were carried out in PCR tubes. The reaction was set up using different primers and 80–100 ng of total DNA of each sample was used as a template for amplification. These included F3 and B3 primers at 0.2 µM each, FIP and BIP primers at 1.6 µM each, and LoopF and LoopB primers at 0.4 µM each. Eight units of the WarmStart *Bst* polymerase (large fragment; New England Biolabs, Ipswich, MA, USA) were used for amplification. Other chemicals including 1.4 mM dNTPs, 6.0 mM MgSO_4_, and 1× supplied Isothermal Amplification Buffer (20 mM Tris-HCl, 10 mM (NH_4_)_2_SO_4_, 50 mM KCl, 2 mM MgSO_4_, and 0.1% Tween 20, pH 8.8) were also used in the reaction. Finally, the crab DNA was added to the reaction mixture as designed. A Mastercycler Nexus thermocycler (Eppendorf, Hamburg, Germany) was employed for LAMP amplification at 68 °C for 30 min, followed by the inactivation of the reaction at 80 °C for 5 min. All LAMP products were detected using 2% agarose gel electrophoresis before staining using 1 µg/mL ethidium bromide, followed by fluorescence illumination. The negative control was performed and checked in all amplifications of each primer.

### 2.5. Polymerase Chain Reaction (PCR) Reaction

The partial region of the COI gene was amplified in the PCR reaction using the AllTaq Master Mix Kit (Qiagen, Hilden, Germany). The reaction mixtures were set up in a total volume of 20 µL containing the F3 and B3 primers at 0.4 µM each, which were designed from the consensus sequence as the primers in the LAMP methods (Table 1). For the PCR reaction, 80–100 ng of the total DNA of each sample was used as a template for amplification. PCR amplification was carried out as follows: 95 °C, 2 min, followed by 32 cycles of denaturation (95 °C, 5 s), annealing (52 °C, 15 s), and extension (72 °C, 10 s), and a final extension (72 °C, 2 min), in which the reaction was completed. Amplicons were visualized using 2% agarose gel electrophoresis and stained with 1 µg/mL ethidium bromide solution. The negative control was performed and checked in all amplifications of each primer.

### 2.6. Specificity and Sensitivity Testing

The determination of cross-species amplification between each primer was carried out by testing each primer set with DNA extracted from all types of swimming crabs (blue swimming crab, three spotted swimming crab, and crucifix crab). The reaction was set up for both the LAMP and PCR methods. A sensitivity evaluation was also performed to determine the minimal DNA amount in each detection method. The quantity of DNA used included 50, 25, 12.5, 6.25, 3.12, 1.56, 0.78, and 0.4 ng/reaction. The PCR amplicons and LAMP products were visualized by 1 µg/mL ethidium bromide staining and fluorescence illumination after agarose gel electrophoresis.

## 3. Results

### 3.1. LAMP Primer Design and Characteristics

With the aim to identify the adulteration of lower priced crab meat in blue swimming crab meat, two types of swimming crabs were selected to mix with the blue swimming crab meat for adulteration. This is because of their availability and similar meat characteristics such as the meat size and texture. Therefore, the crucifix crab and three spotted swimming crab were chosen.

**Figure 1:**
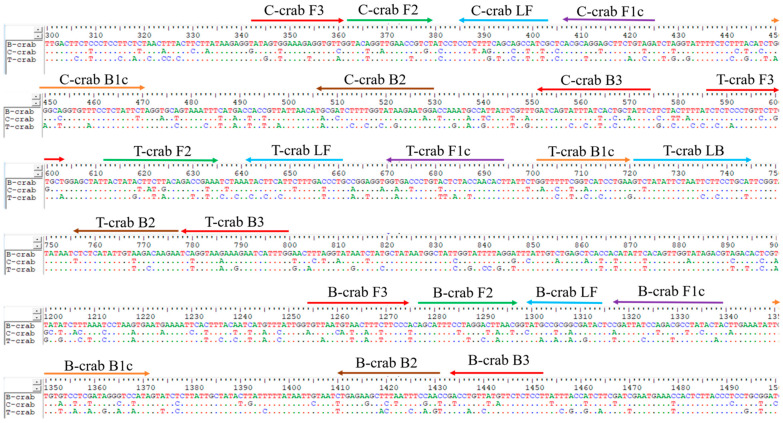
The annealing locations and sequences of the LAMP primers targeting the three types of swimming crabs. The arrows indicate the direction of extension; B-crab—blue swimming crab, C-crab—crucifix crab, T-crab—three spotted swimming crab.

To develop the sets of LAMP primers specific to the three types of swimming crabs, the mitochondrial COI genes of each swimming crab species were retrieved from the GenBank database and aligned using Clustal Omega. The identity of the COI sequence between blue swimming crab, crucifix crab, and three spotted swimming crab was in the range of 82–85% similarity (Table 2). All sequences were subjected to PrimerExplorer V5 online software, yielding the three sets of LAMP primers shown in Table 1. Although the COI genes of all swimming crabs in this study had the same size of the sequence, the LAMP primers designed could cover the different regions of the COI gene for different crab species (Figure 1). Primers in each set contained all the necessary sequences for LAMP amplification including F3, B3, F2, B2, F1c, B1c, and LF, except for the three spotted swimming crab, which had the additional LB primer. The amplification size of the outer primers (F3-B3) was 198 bp, 232 bp, and 214 bp for the blue swimming crab, crucifix crab, and three spotted swimming crab, respectively.

### 3.2. Specific Amplification of LAMP Primers to Different Types of Swimming Crabs

**Figure 2:**
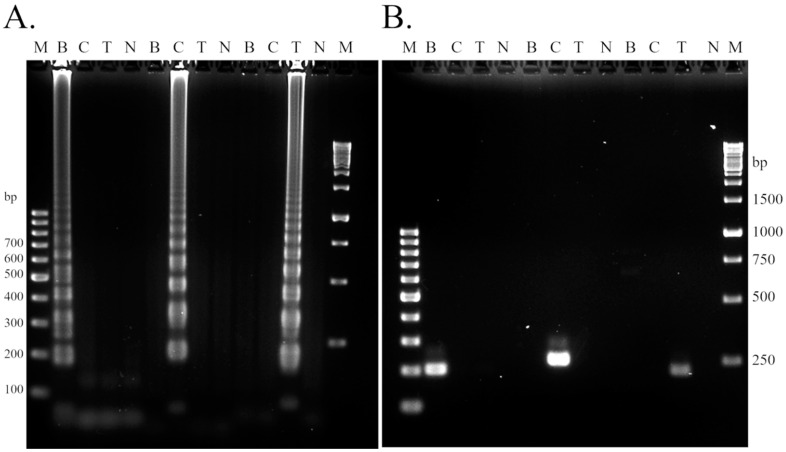
The primer specificity of the LAMP assay for the identification of three types of swimming crabs. The specificity of the COI-based LAMP assay (**A**) and COI-based PCR assay (**B**). M lanes represent the 100 bp and 1 kb DNA ladders. The estimated size of the amplicon was 198 bp, 232 bp, and 214 bp for the blue swimming crab, crucifix crab, and three spotted swimming crab, respectively. B—blue swimming crab, C—crucifix crab, T—three spotted swimming crab, N—negative control.

To evaluate the specificity of LAMP primers, all primer sets were tested with DNA from different swimming crabs. Moreover, PCR amplification using F3-B3 primers was also performed for comparison. The results showed that both LAMP and PCR demonstrated high specificity (Figure 2). For the LAMP test, the ladder forms of the LAMP amplification products were found in agarose gel electrophoresis with the accurate species-specific lane. Primers for the blue swimming crab produced the product when the blue swimming crab DNA was used, and this specificity also occurred with other primers designed for other certain crab species (Figure 2A). In addition, the results from the PCR amplification also provided the same specificity when F3-B3 primers were applied, but it needed a longer time for the reaction compared to LAMP. These results revealed the possibility of the LAMP method for the authentication application of crab meat or products, which could be used to identify three different crab species within a short time (30 min amplification). Therefore, the adulteration could be detected in blue swimming crab meat by other crab meats via different primers.

### 3.3. Sensitivity Testing of LAMP Primers

**Figure 3:**
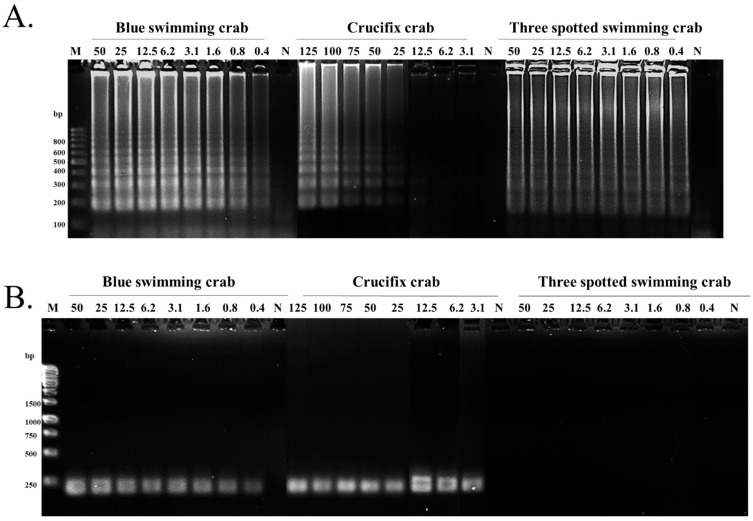
The primer sensitivity test of the LAMP assay for the identification of the three types of swimming crabs. The sensitivity of the COI-based LAMP assay (**A**) and COI-based PCR assay (**B**). M lanes represent the 100 bp and 1 kb DNA ladders. All concentrations of DNA were added to the reaction as nanograms (ng). The estimated size of the amplicon was 198 bp, 232 bp, and 214 bp for the blue swimming crab, crucifix crab, and three spotted swimming crab, respectively. N—negative control.

In addition to the evaluation of the primer specificity, the limits of the detection tests were also elucidated using the different concentrations of DNA extracted from the raw crab meats. The initial amount of DNA was 50 ng/reaction (except crucifix crab) and continued for seven times of 2-fold serial dilution, which ended at 0.4 ng/reaction. Both LAMP and PCR were employed for all three swimming crabs, and agarose electrophoresis was used for product detection. The results demonstrated that all concentrations of blue swimming crab DNA could be detected by both the LAMP and PCR methods in a quantity dependent manner (Figure 3). However, the LAMP products using the DNA of the crucifix crab were observed only when DNA at 50 ng was applied. Overall, PCR provided the obvious amplicons at all DNA amounts (Figure 3B). On the other hand, LAMP showed good sensitivity in all concentrations of DNA isolated from the three spotted swimming crab. Nevertheless, PCR reactions using the F3-B3 primers did not result in any product detected by agarose gel electrophoresis (Figure 3). These results indicate that the LAMP method could be used for blue swimming crab and three spotted swimming crab at a low quantity of DNA, even at 0.4 ng, while DNA above 50 ng of crucifix crab was needed for clear results.

### 3.4. Application of the LAMP Assay in Cooked Swimming Crabs

**Figure 4:**
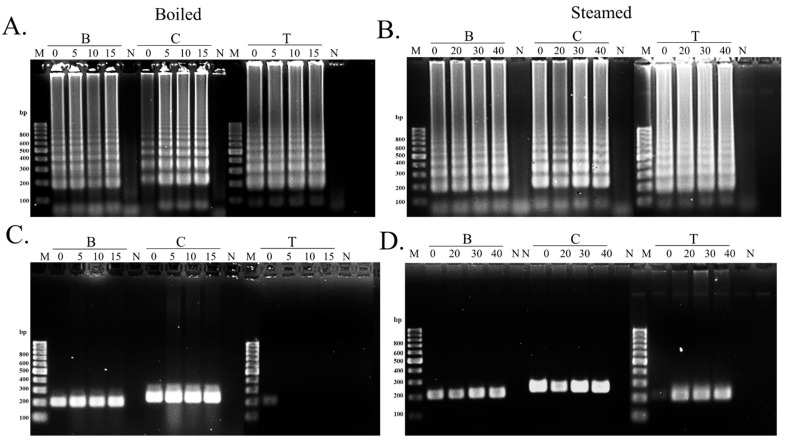
The analysis of the LAMP (**A**,**B**) and PCR (**C**,**D**) products derived from the DNA extracted from different types of cooked swimming crabs. For the boiled sample, the crab meat was cooked for 5, 10, and 15 min. For the steaming process, crab samples were treated for 20, 30, and 40 min before collecting the cooked meat. The estimated size of the amplicon was 198 bp, 232 bp, and 214 bp for the blue swimming crab, crucifix crab, and three spotted swimming crab, respectively. M lanes represent the 100 bp ladder. B—blue swimming crab, C—crucifix crab, T—three spotted swimming crab, N—negative control.

For real-life applications, crab meat is usually cooked before consumption to enhance its safety. Therefore, the determination with the aid of LAMP in cooked crab meat was performed in both boiled and steamed meat for different times (5, 10, and 15 min). When the same concentration of DNA from different crab meats was applied to LAMP and PCR, the detection capacity of LAMP products derived from the boiled blue swimming crab meat and crucifix crab meat was similar to that of PCR, regardless of the boiling time. Thus, boiling times did not have an influence on the effectiveness of the LAMP and PCR detection of blue swimming crab and crucifix crab (Figure 4). However, LAMP provided a better performance than the PCR method for three spotted swimming crab meat since all boiling times did not affect the LAMP reaction, but no amplification product was observed in the PCR reactions after boiling. Whole-body steaming at 20, 30, and 40 min of all swimming crabs was also conducted prior to meat collection. The lump meats were selected for DNA isolation. The results demonstrated that both LAMP and PCR could detect and identify blue swimming crab and crucifix crab, irrespective of the steaming times. For the three spotted swimming crab meat, the LAMP technique showed a better intensity of the DNA band than that of PCR at 0 min. These results indicate that LAMP is the preferred method for cooked samples since all heating times did not affect the LAMP performance for the meat sample detected.

## 4. Discussion

Mislabeling, misrepresentation, or adulteration are common deceptions that are generally found in the seafood market with the intentional substitution for economic benefit. Crabs are one of the targets for food fraud because of their high demand and commercial value worldwide [7]. The blue swimming crab is the dominant species used for canned crab meat products. Because of its high price, the substitution of crab meat with a lower value often occurs and is falsely sold as blue swimming crab at a premium price. In this study, two swimming crabs including three spotted swimming crab and crucifix crab, were selected as the low-price targets. These species are naturally co-distributed with blue swimming crab in the coastal areas of the Indo-Pacific region [21,22]. Moreover, their cooked meat has quite a similar appearance and texture to blue swimming crab meat. This leads to difficulty in species identification and may lead to product misrepresentation. For this reason, a DNA-based authentication method was developed in this study to accurately verify crab products and solve crab meat mislabeling.

DNA barcoding is a molecular technique that is widely used for species identification and food authentication [10]. Although there are several loci in DNA regions that can be applied for the detection of mislabeled foods, the mitochondrial COI gene was selected in this study. The superior advantages of using COI include a large number of COI sequences available for a variety of marine species, the lack of intron, high copy number, and conservative sequence between species [23]. The success in using the COI gene for seafood authentication or misrepresentation detection has been documented. Harris et al. (2016) employed the COI gene for mislabeled seafood detection, and they found that approximately 19% of the examined samples were misrepresented products, especially crustaceans and bivalves [24]. Another study also used this gene to differentiate crustaceans and shrimp from commercial sources mediated by PCR amplification and phylogenetic analysis [25].

In the present study, the highly effective LAMP method has been applied for swimming crab meat diagnosis. High discrimination ability among meats from blue swimming crab, crucifix crab, and three spotted swimming crab was confirmed, although the sequence of the COI gene between these crabs showed a 82–85% similarity. One of the benefits of using LAMP for food authentication is that less effort of optimization for specificity is required since the reaction is mediated by 2–3 pairs of primers recognizing up to eight certain regions on the DNA target [26,27]. This is different from PCR, which uses a pair of primers for target amplification, making PCR tend to show a low specificity if close species are used [28].

An unexpected result was found in the sensitivity test, in which PCR showed a better result than LAMP after various concentrations of crucifix crab DNA were used in the reaction. This might be due to the effect of the low integrity of the DNA template. The more the primers in the LAMP reaction, the higher the quality and integrity of the template required [29,30]. Moreover, seafood has been reported to contain some types of *Bst* polymerase inhibitors such as cationic polysaccharides and/or calcium ions that inhibit polymerase activity and competitively bind to *Bst* polymerases with magnesium ions [31]. Therefore, a DNA extraction method developed for food samples is required to obtain high-quality DNA [32]. LAMP demonstrated a superior capacity in three spotted swimming crabs, while PCR could not detect the DNA at any concentrations. Conversely, LAMP provided the products at all amounts of DNA used. This was in accordance with a previous study that demonstrated that LAMP primers in the reaction increased the sensitivity of detection compared to conventional PCR and multiplex PCR [33]. Based on the detection limit of LAMP, this method exhibited similar analytical sensitivity to quantitative real-time PCR [34].

Heat treatment processes such as boiling and steaming are usually applied to crab meat or crab products before consumption or distribution. This study revealed that heat did not affect the sensitivity of LAMP after the DNA isolated from boiled and steamed crab meat was employed. Although LAMP showed a lower sensitivity than PCR for crucifix crab in the sensitivity test, it was not problematic when the DNA from cooked samples was used. In this case, the concentration and integrity of the DNA template might be factors for the different quality of amplification [30]. Indeed, some reports have described that the increase in treatment temperature led to a higher degradation of DNA in the meat [35,36]. However, no research article has directly compared the DNA quality isolated from boiled and steamed crab meat. In contrast, many works have revealed the greater negative effect of boiling on nutritional value, phenolic content, or the quality characteristics of the food compared to the steaming method [37,38,39]. This might imply that boiling may be more destructive to the meat structure at molecular levels while steaming may be less deleterious, since this may cause the forming of coating surface proteins during high temperature that prevent the escape of moisture [37]. Therefore, a coating by a surface protein might protect the loss of DNA stabilizing substances that could be washed out together with the water during the boiling. As demonstrated by another study, steaming provided a greater retention of antioxidants compared to the boiling method in food [38]. Another report also mentioned that crustaceans could synthesize and accumulate astaxanthin, a carotenoid with potent antioxidant activity, in their tissues [40]. Therefore, steaming plausibly prevented the loss of astaxanthin more effectively than the boiling method. As a consequence, it might help to reduce DNA degradation mediated by reactive oxygen species (ROS), which could be found in meat subjected to heat. These active species are able to attack the cellular compositions including DNA [41,42]. Consequently, the astaxanthin retained might exhibit the preventive effect against ROS-induced DNA damage [42,43].

## 5. Conclusions

The authentication method based on the DNA-barcoding technique was successfully developed to detect adulteration among the meat from three types of swimming crabs. This isothermal condition-based DNA amplification is a specific, sensitive, and rapid identification method and was proven to be a useful tool for the verification of the cooked meat of those three crabs. This developed assay demonstrated superior advantages over the standard conventional PCR since a thermocycler is not necessary, and the reaction can be performed in isothermal conditions. The further application of this technique for the routine inspection of crab meat misrepresentation or mislabeling can be developed by government authorities.

## Figures and Tables

**Table 1:** The primers used in this study.

Primers *	Sequences (5′->3′)
Blue swimming crab F3 B3 FIP (F1c-TTTT-F2) BIP (B1c-TTTT-B2) LF	TGTTAATGTAACTTTCTTCCCA AGGAGAGAACATAACAGGTC AGTAGTATAGGCGTCTGGATAATCGTTTTAGCATTTCCTAGGACTTAACG ATTGTGTCCTCGATAGGGTCCATATTTTGTTGGAAATTAAAGCTTCTCAG GTATCGCCGCGGCATA
Crucifix crab F3 B3 FIP (F1c-TTTT-F2) BIP (B1c-TTTT-B2) LF	GGATAGTTGAAAGAGGTGTCG GAATTGCGGTAATAAATACTGATC TCAACAGAAGCACCTGCGTGTTTTGTACTGGATGAACCGTGT CTGGCCGGTGTTTCCTCTATTTTTTTTTCTATTCTTATACCAAAAGAGCGTA CAATAGCGGCTGCTAAAGG
Three spotted swimming crab F3 B3 FIP (F1c-TTTT-F2) BIP (B1c-TTTT-B2) LF LB	TCCCTACCTGTTCTTGCA CCAAATGATTCCTTTTTACCAGA AGTGTTGATATAAAACAGGGTCTCCTTTTTACTATGCTTTTAACAGATCGTAAC GGTTCTTTGGCCACCCTGAGTTTTTTCTTGGCTAACAATATGAGAG CAGGATCAAAGAAGGAGGTGT GTCTATATTCTAATCCTCCCTGCTT

* Poly T linker was added to the FIP and BIP between F2-F1c and B2-B1c to improve the loop formation and reaction speed.

**Table 2:** The percent identity matrix of the COI gene of different swimming crabs.

Crab Names	Percent Identity (%)		
	Three Spotted Swimming Crab	Blue Swimming Crab	Crucifix Crab
Three spotted swimming crab	100.00	83.31	82.01
Blue swimming crab	83.31	100.00	85.14
Crucifix crab	82.01	85.14	100.00

## Data Availability

The data presented in this study are available on request from the corresponding author.
